# A Review of Models for Heat Transfer in Steel and Concrete Members During Fire

**DOI:** 10.6028/jres.126.030

**Published:** 2021-10-22

**Authors:** Dilip K. Banerjee

**Affiliations:** 1 National Institute of Standards and Technology, Gaithersburg, MD 20899, USA

**Keywords:** concrete members, fire, heat transfer analysis, steel members

## Abstract

Structural design for fire is conceptually similar to structural design conducted under ambient temperature conditions. Such design requires an establishment of clear objectives and determination of the severity of the design fire. In the commonly used prescriptive design method for fire, fire resistance (expressed in hours) is the primary qualification metric. This is an artifact of the standard fire tests that are used to determine this quantity. When conducting a performance-based approach for structural design for fire, it is important to determine structural member temperatures accurately when the members are exposed to a real fire. In order to evaluate the fire resistance of structural members such as structural steels and concrete, both the temporal and spatial variation of temperatures must be accurately determined. The transient temperature profiles in structural members during exposure to a fire can be determined from a heat transfer analysis. There are several models/approaches for analyzing heat transfer that have been used to determine the transient structural temperatures during a fire event. These range from simple models to advanced models involving three-dimensional heat transfer analysis employing finite element or finite difference techniques. This document provides a brief summary of some of the common simple and advanced approaches that have been used for conducting heat transfer analysis of both steel and concrete members when exposed to fire.

## 1 Introduction

The thermal response of structural members during exposure to a fire can be determined by using the principles of heat transfer. The goal of the heat transfer analysis is to determine both the temporal and spatial variation of temperatures. Such information can be helpful to determine if any predetermined critical temperature has been attained during fire exposure. It may be mentioned here that reliance on predetermined critical temperatures (or postulations of structural performance) and/or use of the standard fire curve are/is not proper when employing structural fire engineering. Temperatures determined from heat transfer analysis are used for conducting subsequent structural analyses. Note that the exposure of a structure to fire can be described by subjecting the structural member to the standard fire (time-temperature curve) (*e.g.*, ASTM E119, International Organization for Standardization [ISO] 834 [1, 2]) or an actual fire curve developed through experiments.

When conducting a performance-based approach for structural design for fire, it is important to determine member temperatures accurately when the members are exposed to a real fire event. The relevance of a performance-based approach in the context of fire safety assessment of long tunnels has been outlined in Ref. [3]. Heat is transferred from hot gases in fire to the fire-exposed surfaces of structural members through both convection and radiation. Additionally, conductive heat transport is instrumental in transferring heat to adjacent members. At elevated temperatures, radiation plays a more dominant role in transferring heat to members. The heat that is transferred to the exposed surfaces of members diffuses within the members, mainly by the heat conduction mechanism. This is true for members that are considered to be homogeneous. Mathematically, this transfer of heat is handled using proper boundary conditions. If there are voids within the members or the members are porous, then both convection and radiation can influence the heat transfer within the members. For the sake of simplicity, only conductive heat transfer approaches are described in this document. It may be noted that errors associated with non-inclusion of voids are insignificant if the void fractions are relatively small. Appropriate contact thermal resistances at the interfaces of dissimilar materials must be considered in a heat transfer analysis. Heat transfer in members exposed to fire can be described using approaches that range from simple analytical equations to advanced models such as finite element analysis (FEA) models.

A coupled thermal-structural analysis can provide an accurate insight into the behavior of a structural assembly during fire. However, such analysis is computationally quite challenging and often prohibitive. Hence, researchers have sought to decouple the calculation of thermal response from the determination of structural response, provided the structural geometry does not suffer significant changes in overall dimensions [4]. This is often appropriate for steel structures since they are generally protected by thermal insulation. However, this assumption is not valid when there is total loss of protection. On the other hand, such a decoupled approach is often difficult when applied to concrete, since spalling is a phenomenon that affects the boundaries of concrete cover. This can expose reinforcement to fire, leading to a large loss of strength. However, since the mechanism of spalling is not completely understood in terms of a mathematical implementation in a model, the decoupled approach is still the best option [4].

While reviewing the literature on heat flow during fire gaps were found in the information that is needed for engineers to make their structural fire design calculations more accurately. Some of the traditional techniques (which are available in engineering heat transfer literature) of simple heat flow calculations have not been widely adopted by the structure-fire community. This document attempts to address this deficiency. In this document, various approaches that can be used for determining temperatures of both steel and concrete members during fire exposure are discussed. Common approaches for computing heat transfer during fire in steel and concrete structures are discussed. However, the focus is on discussing simplified approaches for estimating member temperatures in fire. For this purpose, the boundary conditions and governing equations of heat flow in fire need to be addressed. Hence, this document will briefly describe the common boundary conditions and governing equations used in fire protection engineering, after which approaches for estimation of heat flow in steel and concrete members will be discussed. Simple approaches for heat flow in concrete-filled steel tubes (CFSTs) are also briefly discussed.

On completing this tutorial, readers will have learned how to determine structural member (steel, concrete, *etc*.) temperatures when these members are exposed to a real fire event. Such information is very important for the determination of structural behavior under both fire and mechanical loads.

### 1.1 Audience

This tutorial is designed to be used by structural-fire engineers in a typical design office and those students that intend to pursue graduate-level research after some industrial experience.

### 1.2 Education or Skill Level

Readers of this tutorial should be familiar with the fundamental knowledge of heat transfer. In addition, they should have some general understanding of structural behavior during fire. Some knowledge of building structure assemblies is desirable.

### 1.3 Prerequisites

The reader should have sufficient knowledge of building structures and standard fire tests (ASTM E119, ISO 834 [1, 2], *etc*.). Some familiarity with FEA software and analytical solution techniques for partial differential equations is needed.

### 1.4 Tools or Equipment

Equipment includes desktop or laptop computer equipment, general-purpose FEA computer programs, or special-purpose computational software for structural-fire analyses.

### 1.5 Background

In performance-based design of structures for fire, accurate estimation of time-dependent temperatures of structural members are needed for the determination of structural behavior during fire. This tutorial is focused on discussing various heat transfer modeling approaches (both simplified and comprehensive) for determination of member temperatures during fire. Accurate determination of these temperatures is vital to determine how a building assembly will behave during a real fire event. In this tutorial, first a brief description of the boundary conditions in fire is provided, which is followed by a discussion on the governing equations of heat flow in fire. Then, common approaches for determination of temperatures of steel, concrete, and CFSTs are described.

Boundary conditions in fire are needed to conduct a heat transfer analysis in order to determine member temperatures during the fire event. The governing equations are used to describe heat flow along with initial and boundary conditions. The three common boundary conditions that are used in fire protection engineering are: (1) prescribed surface temperature (or Dirichlet condition), shown in Eq. (1.1), (2) prescribed surface heat flux (or Cauchy condition), as in Eq. (1.2), and (3) natural boundary condition (or Neumann condition), given in Eq. (1.3). There are three variants of the natural boundary conditions. They are: (a) natural boundary condition (prescribed convective heat transfer), (b) prescribed convection and radiation assuming equal radiation and gas temperatures, and (c) prescribed convection and radiation assuming different radiation and gas temperatures. The mathematical expressions for these boundary conditions are expressed below:

$T_{x=0}=T_{s}$ (1.1)

$-k{\left.\frac{\partial T}{\partial x}\right|}_{x=0}={\dot{q}}_{s}^{'}\;(1.2)$

$-k{\left.\frac{\partial T}{\partial x}\right|}_{x=0}=h\;\left(T_{g}-T_{s}\right)\;(1.3a)$

$-k{\left.\frac{\partial T}{\partial x}\right|}_{x=0}=h_{c}\;\left(T_{f}-T_{s}\right)+\epsilon \;\sigma \;\left(T_{f}^{4}-T_{s}^{4}\right)\;(1.3b)$

$-k{\left.\frac{\partial T}{\partial x}\right|}_{x=0}=h_{c}\;\left(T_{g}-T_{s}\right)+\epsilon \;\sigma \;\left(T_{r}^{4}-T_{s}^{4}\right)\;(1.3c)$

$-k{\left.\frac{\partial T}{\partial x}\right|}_{x=0}=h^{AST}\left(T_{AST}-T_{s}\right)\;(1.3d)$

where *T_x=0_* is the member surface temperature, *k* is the thermal conductivity, *h* is the heat transfer coefficient, *h_c_* is the convective heat transfer coefficient, $h^{AST}$ is the adiabatic heat transfer coefficient, *T*_AST_ is the adiabatic surface temperature, *ε* is the emissivity, *σ* is the Stefan-Boltzmann constant, *T_g_* is the gas temperature, *T_s_* is the member surface temperature, *T_f_* is the fire temperature, ${\dot{q}}_{s}^{'}$ is the heat flux, and *T_r_* is the radiation temperature.

According to Wickstrom [5], the first and second boundary conditions are sparingly used in fire protection engineering applications. In many cases, heat transfer coefficients, *e.g.*, Eq. (1.3a), and emissivities, *e.g.*, Eqs. (1.3b) and (1.3c), are assumed to be constant, although they vary as a function of temperature in reality. Equations (1.3b) and (1.3c) are commonly used. These equations consist of a convective term and a radiative term. Equation (1.3b) assumes that the radiation temperature is equal to the gas temperature. Equation (1.3b) is applied when using time-temperature design curves according to ISO 834 or ASTM E119 [1, 2], where the fire temperature is given by the standard fire. Equation (1.3c) allows the use of different gas and radiation temperatures. Both Eqs. (1.3b) and (1.3c) can be expressed in the form of Eq. (1.3a) with an effective heat transfer coefficient, *e.g.*, *h^AST^* in Eq. (1.3d). Wickstrom [5] suggested that a single effective temperature (*i.e.*, adiabatic surface temperature, *T_AST_*) can be defined with a value between the radiation and gas temperature. By definition, *T_AST_* is the temperature of a surface that cannot absorb any heat and is typically a weighted value of the radiation and the gas temperature and therefore is dependent on both the surface emissivity, *ε*, and the convective heat transfer coefficient, *h_c_*. The radiation, gas temperatures, and the adiabatic surface temperatures vary as a function of time during a fire event. The *h^AST^* and *T_AST_* terms can be expressed by the following equations ($h_{c}^{AST}$ is assumed to be the same as *h_c_*) [5]:

$h^{AST}=h_{r}^{AST}+h_{c}^{AST}\;(1.3e)$

$h_{r}^{AST}=\epsilon \;\sigma \left(T_{AST}^{2}+T_{s}^{2}\right)\left(T_{AST}+T_{s}\right)\;(1.3f)$

$T_{AST}=\frac{h_{r}T_{r}+h_{c}T_{g}}{h_{r}+h_{c}}\;(1.3g)$

$T_{r}=\sqrt[{4}]{\frac{{\dot{q}}_{inc}^{'}\;}{\sigma }}\;(1.3h)$

where ${\dot{q}}_{inc}^{'}$ is the incident radiation flux. The convection heat transfer coefficient remains the same because it is assumed to be independent of the exposure temperature ($h_{c}^{AST}$= *h_c_*). Note that Eq. (1.3g) needs to be solved iteratively because *h_r_* depends on *T_AST_*. Detailed information about these expressions is described by Wickstrom [5].

In order to compute the thermal response of a structural member, one must consider two aspects of heat flow: (1) the heat transfer from the boundary of the furnace or fire to the outer surface of a member by a combination of convection and radiation (treated as boundary conditions) and (2) subsequent heat transfer within a structural member *via* conduction (treated as governing equations) following Fourier’s equation of heat conduction. This equation is given as:

q= -k∇T (1.4a)

where *q* is the heat flux vector, *k* is the thermal conductivity, and *T* is the temperature. Then, the equation for the conservation of energy can be expressed as:

$\rho C_{p}\frac{\partial T}{\partial t}=\;-\;\nabla \;.\;q+\;\dot{\dot{Q.}\;(1.4b)}$

where *ρ* is the density, *C_p_* is the heat capacity, *t* is time, and $\dot{Q}$ is the heat source term. Equation (1.4b) is solved with initial conditions and proper boundary conditions. Convective heat transport is due to the motion of the fluid and depends on surface geometry, nature of fluid motion, fluid properties, and fluid viscosity [4]. EN 1991-1-2 recommends convective heat transfer coefficient values of 25 W/m^2^/K and 9 W/m^2^/K for a fire-exposed surface (with standard fire) and an ambient exposed surface, respectively. On the other hand, radiative transport entails transfer of thermal energy by electromagnetic waves that do not require any medium. In order to account for varying radiative heat flux levels (while keeping both surface and fire emissivities at constant levels), a configuration factor, *Φ*, is introduced in radiative heat flux expressions. In fire protection engineering, the value for this configuration factor is conservatively taken as 1. In the expressions in Eq. (1.3b) and Eq. (1.3c), *Φ* is taken as 1. Drysdale [6] gives more details about calculations of this configuration factor.

## 2 Instructions

In this section, both simplified and comprehensive approaches for determination of the heat flow in structural members during fire will be described. First, commonly used approaches for determination of steel member temperatures in fire will be described, and this will be followed by the approaches for concrete members and CFSTs.

### 2.1 Steel

Both simplified and comprehensive approaches can be used to determine temperatures in steel beams and columns during fire exposure.

#### 2.1.1 Simplified Approaches for Steel

Simplified approaches include the following:

• Lumped mass approach for unprotected steel
• Lumped mass approach for steel with low-heat-capacity insulation
• Lumped mass approach for steel with high-heat-capacity insulation
• EN 1993-1-2 approach
• Best-fit method for steel with insulation
• Graphical solutions

##### 2.1.1.1 Lumped Mass Approach for Unprotected Steel

The “lumped mass” or “lumped heat capacity” method assumes that there is no temperature gradient in a member (*e.g.*, member temperatures are uniform). This is an idealized case because in reality a temperature gradient is present in a member when heat conducts into or out of a body.

In general, this assumption of a uniform temperature throughout the member is more realistic for smaller members and larger values of thermal conductivity. The method is valid if the following inequality is maintained [7]:

$\frac{h\;(\frac{V}{F})}{k}$ ﹤0.1 (2)

where *h* is the total heat transfer coefficient, *k* is the thermal conductivity, *V* is the volume, and *F* is the surface area of the member. The left-hand side of Eq. (2) is also called the dimensionless Biot number. This number plays a fundamental role in conduction problems that involve heat exchange with the environment. The Biot number provides a measure of the temperature change in the solid relative to the temperature difference between the surface and hot gases. Steel members with typically high thermal conductivity (*k*) are suitable candidates for the use of this method for computing temperatures.

The following heat balance equation is used to derive a simple expression for the change of member temperature. The assumption is that the heat entering in a small time increment is totally used to raise the steel temperatures with no heat loss:

Heat entering = heat used to raise temperature

${\dot{q}}^{"}F\Delta t\;=\;\rho_{s}c_{s}V\Delta T_{s}\;\;\;\;\;\;\;\;\;\;\;\;\;\;(3)$

where ${\dot{q}}^{"}$is the heat flux at the surface (W/m^2^), Δt is the time increment, $\Delta T_{s}$ is the change in steel temperature, $\rho_{s}$ is the steel density, and $c_{s}$ is the steel volumetric heat capacity. Since the heat enters by convection and radiation, Eq. (3) can be rearranged as follows:

$\Delta T_{s}\;=\;\frac{F}{V}\frac{1}{\rho_{s}c_{s}}\left[h_{c}\left(T_{f}-T_{s}\right)+\sigma \varepsilon \left(T_{f}^{4}-T_{s}^{4}\right)\right]\Delta t\;\;\;\;(4)$

where $h_{c}$ is the convective heat transfer coefficient, σ is the Stefan-Boltzmann constant, ε is the emissivity, $T_{f}$ is the gas temperature in fire environment, and $T_{s}$ is the steel temperature. The first and second terms in the square bracket of the right side of Eq. (3) represent convective and radiative heat transfer, respectively.

Spreadsheets are often used for calculating steel temperatures for a certain fire exposure. Gamble proposed such a method [8], where he suggested a time increment of 5 min. *Eurocode 3* (EC3) [9] suggested a maximum time step of 30 s and a minimum section factor value (*F*/*V*) of 10 m^−1^. Kay [10] reported a very good prediction of steel temperatures in standard fire resistance tests.

##### 2.1.1.2 Lumped Mass Approach for Steel with Low-Heat-Capacity Insulation

The calculation approach for protected steel is similar to the one discussed above for unprotected steel. The rate of temperature rise in a protected steel member during fire exposure depends on the thermophysical properties of the steel member (*e.g.*, density, heat capacity, and thermal conductivity) and the rate of heat diffusion through the insulation surrounding the steel member. If it is assumed that there is no temperature gradient in steel, when Eq. (2) holds, steel temperature rise will be governed solely by its heat capacity. Under equilibrium conditions, heat transmitted through insulation should equal the heat used to raise steel temperatures. Assuming that there is no resistance to heat transfer at the exposed surface (*e.g.*, the exposed surface temperature is equal to the fire temperature) and that the thermal heat capacity of insulation is negligible, then the following heat balance equation can be written:

$\rho_{s}c_{s}V\frac{\Delta T_{S}}{\Delta t}=\;k_{i}A_{i}\frac{\left(T_{f}-T_{s}\right)}{d_{i}}\;\;\;\;\;\;\;\;\;\;\;\;\;\;(5)$

where $k_{i}$,$\;d_{i}$,$\;A_{i}$, and$\;T_{f}$ are the insulation thermal conductivity, insulation thickness, surface area of insulation, and fire temperature, respectively. Then, the change in steel temperature is given by:

$\Delta T_{S}=\;{\left(\rho_{s}c_{s}V\right)}^{-1}\left(\frac{k_{i}}{d_{i}}\right)A_{i}\left(T_{f}-T_{s}\right)\;\Delta t\;\;\;\;\;\;\;\;\;\;\;(6)$

The above expression assumes that the temperature distribution through the insulation is linear. The European Commission for Constructional Steelwork (ECCS) [11] suggests that this approximation for low-heat-capacity insulation is valid when the following inequality holds (note that the heat capacity of a material is not necessarily correlated with its weight):

$\rho_{s}c_{s}F\;>\;2\rho_{i}c_{i}A_{i}\;\;\;\;\;\;\;\;\;\;\;\;\;\;\;\;\;(7)$

This is essentially the condition for the lumped mass approach for low-heat-capacity insulation. Note that the time step should be chosen carefully. The accuracy of the computed results is enhanced for smaller time steps. It may be mentioned here that there are situations where absorption of heat may only occur over a specific temperature bandwidth (*e.g.*, endothermic spike). These considerations are not included in the discussion of this section.

##### 2.1.1.3 Lumped Mass Approach for Steel with High-Heat-Capacity Insulation

For steel members with heavyweight insulation, Eq. (7) does not hold. This is often the case for gypsum plaster, masonry, and concrete fire protection systems. In this case, the heavyweight insulation absorbs so much heat such that the amount of heat that is transferred to steel is reduced. This is true when the heating occurs under transient conditions. Therefore, one must consider that the heat that is transferred from fire is used to heat both steel and the insulation. If it is assumed that the temperature at the exposed surface of insulation is the same as the true gas temperature in the fire, then the following expression can be written (heat transfer coefficients are not required in this approach):

$\rho_{s}c_{s}V\frac{\Delta T_{S}}{\Delta t}+\frac{\rho_{i}c_{i}d_{i}A_{i}}{2}\frac{\left(\Delta T_{f}+\Delta T_{s}\right)}{\Delta t}=k_{i}A_{i}\frac{\left(T_{f}-T_{s}\right)}{d_{i}}\;\;\;\;\;\;\;\;(8)$

where $\rho_{i}$ is the density of the insulation, and *ΔT_f_* is the change in fire temperature. The above expression assumes that the temperature profile through the insulation thickness is linear throughout the fire exposure duration. This assumption is usually not valid at the initial stage of the fire. However, overall, it is an effect that can be conservatively neglected [8]. Also, it is also assumed that the temperature at the internal surface of the insulation equals that of the steel member. ECCS [11] recommends addition of half of the heat capacity of the insulation to the steel heat capacity in order to simplify the expression for computation of steel temperatures in Eq. (8). The following equation is obtained following algebraic manipulation (assuming *F* = *A_i_* and $\Delta T_{f\;}$= 0):

$\Delta T_{s}\;=\;\frac{F}{V}\frac{k_{i}}{d_{i}\rho_{s}c_{s}}\left[\frac{\rho_{s}c_{s}}{\left(\rho_{s}c_{s}+\left(F/V\right)d_{i}\rho_{i}c_{i}/2\right)}\right]\left(T_{f}-T_{s}\right)\Delta t\;\;\;(9)$

When the volumetric heat capacity of the insulation (product of $\rho_{i}$ × $c_{i}$) is low, both ECCS [11] and Malhotra [12] suggest omitting the term in square brackets in Eq. (9). Then, the expression in Eq. (9) becomes the same as the one obtained for the lightweight insulation, *e.g.*, Eq. (6). EC3 [9] suggests a slightly different expression for Eq. (9), where they recommend using a factor of “3” as opposed to “2” in the insulation term in the denominator to allow for the temperature gradient in the insulation and an extra term for considering the change in fire temperature in a small time increment.

##### 2.1.1.4 EN 1993-1-2 Approach

Both the EN 1993-1-2 [9] and EN 1994-1-2 [14] adopt the approach proposed by Wickstrom [13] as shown below:

$∆T_{s}=\frac{\frac{k_{i}}{d_{i}}}{\rho_{s}c_{s}}\frac{F}{V}\frac{\left(T_{f}-T_{s}\right)∆t}{1+\frac{\varphi }{3}}-\;\left(e^{\frac{\varphi }{10}}-1\right)∆T_{f}\;(10)$

where *F*/*V* is the exposed surface area per unit volume, and *Φ* is defined as:

$\varphi =\frac{\rho_{i}c_{i}}{\rho_{s}c_{s}}d_{i}\frac{F}{V}\;(11)$

EN 1993-1-2 puts a maximum limit of Δ*t* as 30 s, while Wickstrom recommends a different limit [13] and also recommends a time shift at the beginning of heating. The moisture in insulation can slow the rate of the temperature rise and also can cause a temperature change when water is vaporized at 100 °C. The effect of moisture in insulation can be accounted for by including a moisture-dependent expression for the density of insulation. Alternatively, a delay time can be introduced to account for the time needed for the steel to reach 100 °C [9].

##### 2.1.1.5 Best-Fit Method for Steel with Insulation

For bare steelwork, Twilt and Witteveen [15] proposed the following equation for the time of failure:

$∆T_{s}=0.54\left(T_{s}-50\right){\left(\frac{F}{V}\right)}^{-0.6}\;(12)$

where $T_{s}$ is the temperature in steel reached in time *t* (min). This expression only holds for failure time between 10 and 80 min, steel temperature between 400 °C and 600 °C, and *F*/*V* values between 10 and 300 m^−1^. Other expressions for time to failure for protected steelwork have been proposed. Please see Wickstrom [13] and Melinek and Thomas [16].

According to ECCS [11], the following expression can be used to predict approximately the time (in min) that a steel member protected with a light insulation will take to reach a limiting temperature when exposed to the standard fire [1, 2]:

$t\;=\;40\left(T_{lim}-140\right){\left[\frac{d_{i}/k_{i}}{F/V}\right]}^{0.77}\;(13)$

where $T_{lim}$ is the limiting temperature in °C, $d_{i}$is in m, and all other parameters in Eq. (13) are in SI units. According to ECCS, the empirical in Eq. (13) is valid under the following conditions:

(a) *t* (30 min to 240 min), (b)$\;T_{lim}$ (400 °C to 600 °C), (c) *F/V* (10 m^−1^ to 300 m^−1^), and (d) $d_{i}/k_{i}$ (0.1 m^2^K/W to 0.3 m^2^K/W).

For insulation containing moisture, a time delay is added to the time computed from the above equation [17]. Equation (13) is useful in obtaining the time required to obtain a limiting temperature in a steel member. Alternatively, Eq. (13) can be used to determine when an expected temperature will be attained. Buchanan [17] provides a detailed overview of many simplified approaches that are commonly used for computing member temperatures as a result of exposure to fire.

##### 2.1.1.6 Graphical Solutions

Graphical solutions have been extensively used for solving transient heat conduction in homogeneous bodies. They are described for planar, cylindrical, and spherical geometries. Heisler [18] developed such charts, which have been widely used. It is assumed that the member of interest is placed in a medium (fire environment) at constant temperature and that heat is transferred from fire to the member by convection and radiation, which can be described by an equivalent heat transfer coefficient, *h*. However, the limitations of using these charts are (1) use of uniform fire exposure temperature, (2) simple geometry, and (3) computation of an effective heat transfer coefficient, *h*.

**Fig. 1:**
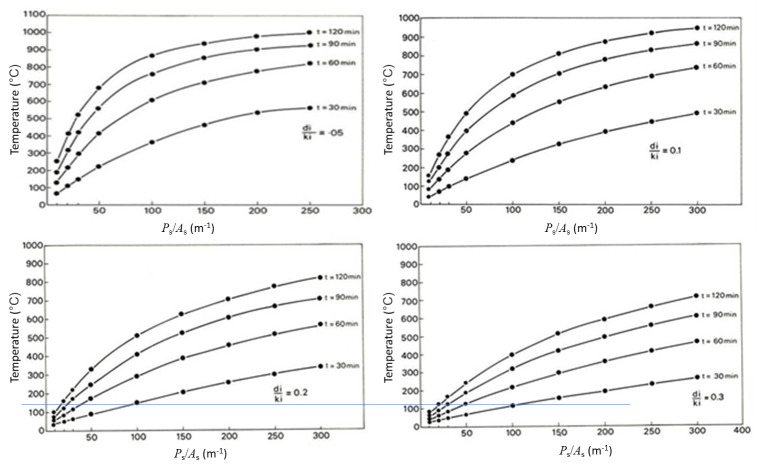
Steel temperatures as a function of shape factor (steel perimeter/area; *P*_s_/*A*_s_) at four times and four different values of the ratio of insulation thickness to insulation thermal conductivity (*d_i_*/*k_i_*; m^2^K/W) during exposure to an ASTM E119 fire. Adapted from [12].

Malhotra, Jeanes, and Lie [12, 19, 20] developed graphs for prediction of temperatures in protected steel members exposed to a standard fire. Malhotra’s graphs were developed using the lumped heat capacity (lumped mass) method, where steel temperatures are plotted as a function of the steel shape factor (steel perimeter/steel area) and at four different values of the ratio of insulation thickness to insulation thermal conductivity for four different times (30 min, 60 min, 90 min, and 120 min into the test) during exposure to the standard (ASTM E119 fire) [1] (Fig. 1).

**Fig. 2:**
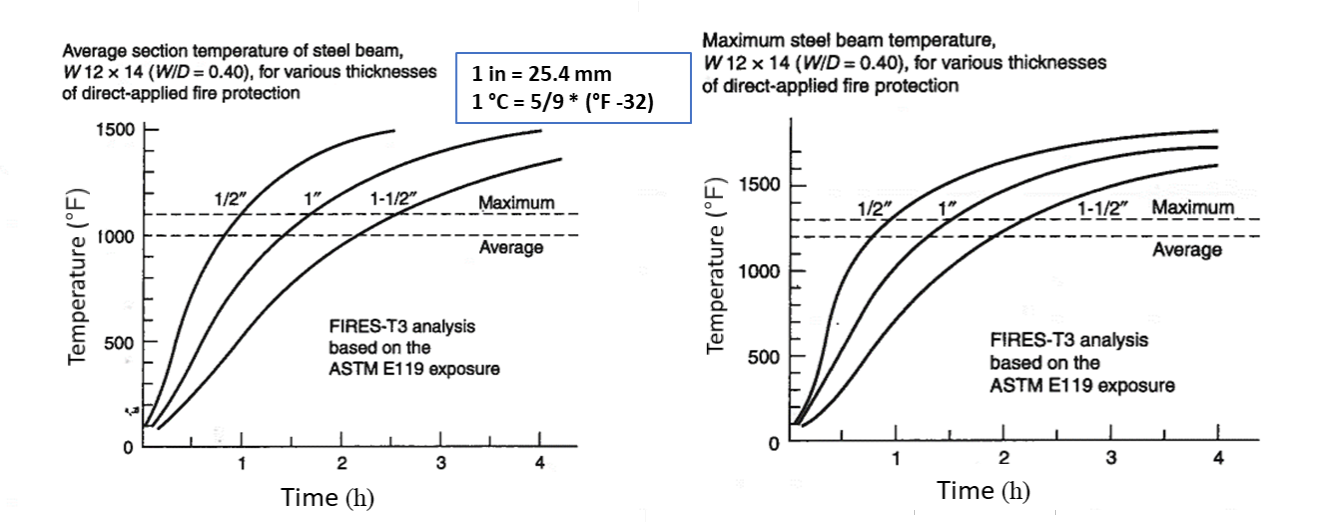
Average section and maximum temperatures of steel beams computed for three different fireproofing thicknesses during ASTM E119 fire exposure using the computer program FIRES-T3 [19] (reprinted with permission).

**Fig. 3:**
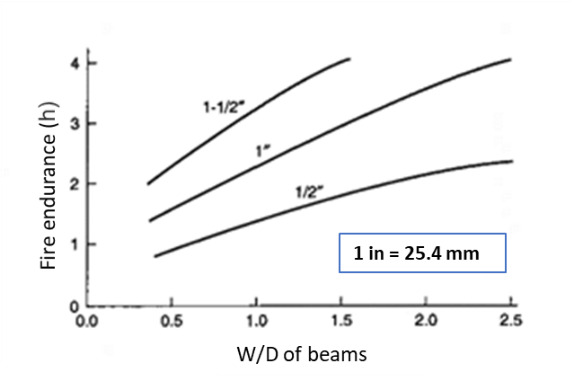
Fire endurance (for average section temperature of 538 °C) as a function of fireproofing thickness computed using FIRES-T3 analysis of exposure to an ASTM E119 fire [19] (reprinted with permission).

Jeanes [19] developed a series of graphs for protected steel beams using a computer program, FIRES-T3 (to be discussed later). These graphs were generated for fireproofing thicknesses between 0.5 in. (1.3 cm) and 1.5 in. (3.8 cm) for most common wide-flange beams. These graphs provide both the average section temperature and maximum temperature of steel beams (Fig. 2). Jeanes [19] provided graphs for determining fire endurance of steel beams as a function of the *W/D* of the beam and fireproofing thickness (Fig. 3). These graphs are valid for steel beams with *W/D* ratios ranging from 0.4 lb/ft-in. (0.023 kg/m-mm) to 2.5 lb/ft-in (0.146 kg/m-mm). Note that *W* is the steel weight per lineal foot, and *D* is the heated perimeter (in).

Lie [20] solved the heat transfer equations and developed plots that show the variation of dimensionless temperatures as functions of the Fourier number and a dimensionless quantity, *N*. *N* is defined as $\rho_{i}c_{i}l/\left(c_{s}M/A\right)$. Here, $\rho_{i}$ and $c_{i}$ are density and heat capacity of the insulation, respectively, and *l* is the length in the direction of heat flow, *c_s_* is the steel heat capacity, *M* is the mass of steel per unit length, and *A* is the area of the interface between fireproofing and steel. The Fourier number (Fo) is the ratio of the heat conduction rate to the rate of thermal energy storage and is mathematically written as $Fo=\alpha t/x^{2}$, where α is the thermal diffusivity, *t* is the characteristic time, and *x* is the length through which heat conduction occurs. For protected steel, the following boundary conditions need to be considered:

At the unexposed surface, the balance of heat flux provides

$h\left(T-T_{0}\right)\;=\;-k\frac{\partial T}{\partial x}\;\;\;\;\;\;\;\;\;\;\;(14)$

**Fig. 4:**
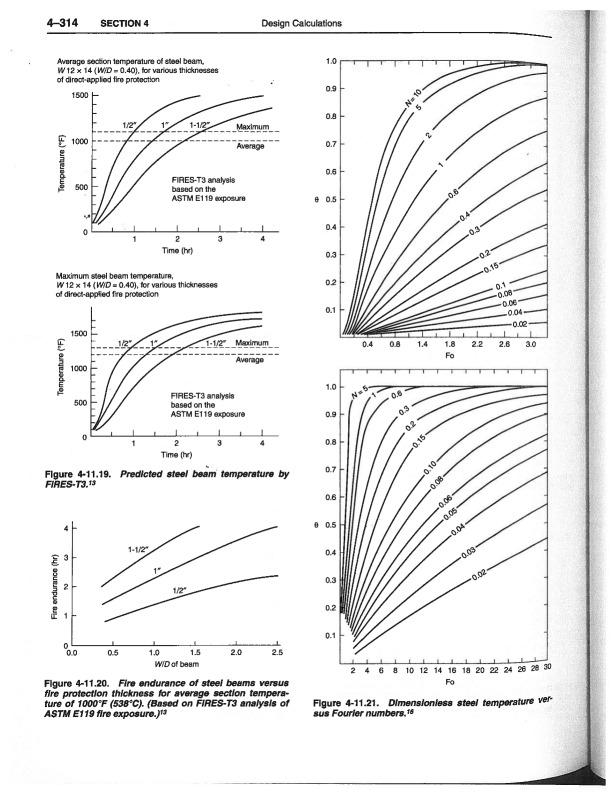
Dimensionless steel temperatures as a function of Fourier number for various types of beam sections (*e.g.*, I section, tubular, *etc*.) [20] (reprinted with permission).

At the steel-fireproofing interface,

$-k\frac{\partial T}{\partial x}=c_{s}\frac{M}{A}\frac{\partial T}{\partial t}\;\;\;\;\;\;\;\;\;\;\;\;(15)$

where *h* is the convective film coefficient at the unexposed surface, *k* is the steel thermal conductivity, and $T_{0}$ is the ambient temperature. The material properties should be computed at each temperature in this method. Thus, the temperature dependence of thermophysical properties of steel can be included. Dimensionless protected steel temperatures for both small and large values of the Fourier number are shown in Fig. 4 [20].

#### 2.1.2 Comprehensive Approaches for Steel

In the performance-based approach, which is increasingly being used to move beyond the prescriptive procedures presently in use, the performance of a structure in actual fire needs to be determined. In this approach, the performance of structural members and systems, including connections, needs to be determined when members are subjected to realistic fires rather than to controlled furnace conditions. Comprehensive approaches for determination of temperatures are more appropriate in a performance-based approach. This is because comprehensive approaches can be used to accurately determine both the temporal and spatial variations of temperatures in members in a real fire.

The computer-based approaches for computing temperatures in steel members during fire exposure range from a simple spreadsheet procedure to advanced, three-dimensional (3-D) finite difference (FD) and finite element (FE) methods. The advanced FE models solve the governing partial differential equations for the conservation of thermal energy—a more general form than that given by Eq. (1.4b)—as given below:

$\rho \;C_{p}\left(\frac{\partial T}{\partial t}+\;\vec{v}\;.\;\nabla T\right)+\;\nabla \;.\;\vec{q}\;=\;\dot{Q}\;(16)$

where $\vec{v}$ is the velocity vector, $\vec{q}$ is the heat flux vector, and $\dot{Q}$ is the heat source term. The advanced models can easily handle temperature-dependent thermophysical properties and temperature/time-dependent boundary conditions and real geometries.

Several heat transfer FE programs have been developed specifically to compute heating of steel structural assemblies exposed to fire conditions. The most popular among them are: FIRES-T3, SAFIR, TASEF-2, HEATING7, and SUPER-TEMPCALC.^1^1 Certain commercial software products or materials are identified to describe a procedure or concept adequately. Such identification does not imply recommendation, endorsement, or implication by NIST that the software products ormaterials are necessarily the best available for the purpose. The input data to these programs can be described as follows:

(1) input of complex geometric information (discretization of structural elements in grids or elements and nodes);
(2) description of thermophysical properties (*e.g.*, density, heat capacity, thermal conductivity, latent heat of fusion/phase change, *etc*.) for steel, insulation, and concrete (if any); and
(3) initial and boundary conditions, including the conditions of fire exposure.

**Fig. 5:**
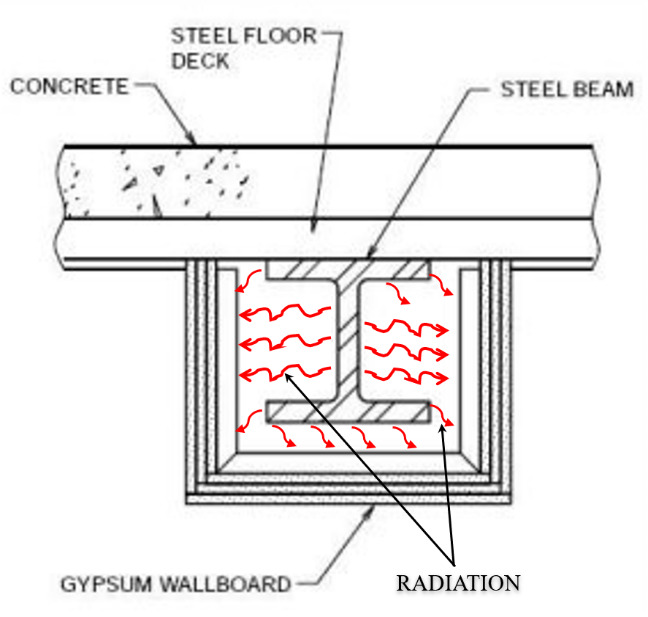
Schematic of cavity radiation.

Harmathy and Lie [21] proposed a two-dimensional (2-D) FD approach to compute temperatures in protected steel columns during fire. Temperatures were assumed to be independent of length. The solution domain was the 2-D cross section of the steel section along with the insulation layer. They considered radiation across any air gap enclosed by the insulation and steel. Convection was neglected in their model. Note that cavity radiation can be important in cases such as when a steel I-beam is boxed in a gypsum board (see Fig. 5).

**Fig. 6:**
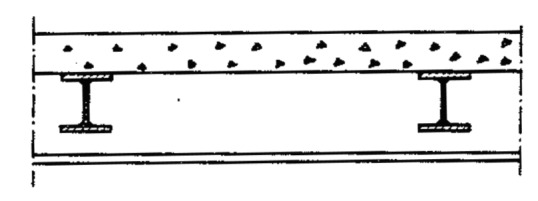
Steel girder floor construction with insulation in the form of a suspended ceiling [22] (reprinted with permission).

**Fig. 7:**
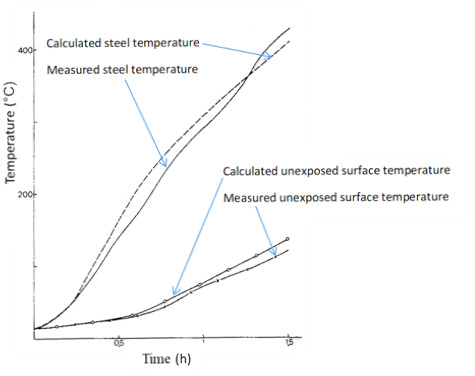
Calculated and measured steel girder temperatures with insulation in the form of a suspended 40 mm thick mineral wool ceiling. Also shown are the calculated and measured temperatures at the unexposed surface of the 50 mm thick concrete floor slab [22] (reprinted with permission).

Pettersson *et al*. [22] published an FD approach for predicting temperatures in steel girders protected with a suspended ceiling that is exposed to fire (see Fig. 6). They proposed a one-dimensional (1-D) approximate solution that accounts for conduction through the suspended ceiling and floor slab and both radiation and convection in the space between the slab and ceiling. Steel temperatures are considered to be uniform. They finish by solving a system of equations for obtaining the temperatures. A comparison of predicted steel column temperatures and measured temperatures is shown in Fig. 7.

TASEF-2 solves heat conduction through assemblies and includes both radiative and conductive heat transfer through internal voids [23]. Both the ISO-834 fire curve and a ventilation-controlled fire curve are included in the software. SUPER-TEMPCALC is similar to TASEF-2 and includes several fire curves [24].

**Fig. 8:**
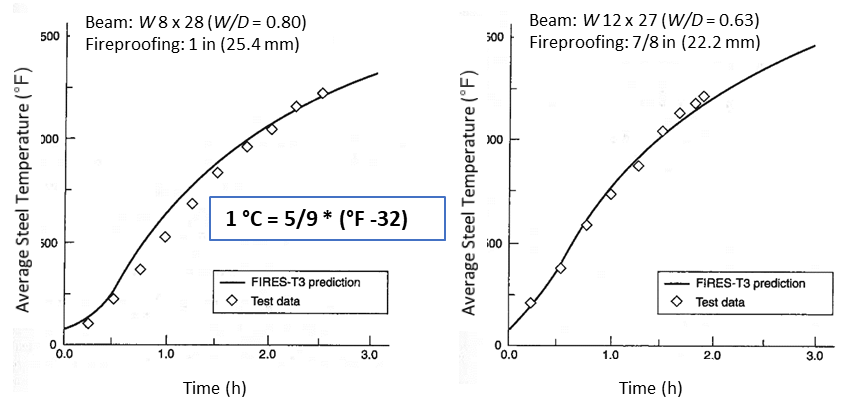
Comparison of experimental data and FIRES-T3 analysis [19]. MONOKOTE (a trademarked material) was used as the fireproofing material (reprinted with permission).

FIRES-T3 has been used to compute temperatures in protected steel beams and columns [25]. Figure 8 shows a comparison of measured temperatures and those computed with FIRES-T3 for a steel beam protected with a spray-applied cementitious material that was exposed to the standard ASTM E-119 fire test.

General purpose FE analysis software is available for modeling the time-dependent temperature distribution in a structure during a fire exposure. These models can be used for a real structure. Temperature-dependent thermophysical properties and both time-dependent and temperature-dependent boundary conditions can be used in such models. Thermophysical properties of different materials in the model can be incorporated in this analysis procedure. Some of the popular commercial software products available include ANSYS, ABAQUS, MSC-NASTRAN, and COMSOL Multiphysics [26–29]. The steps needed to conduct such an analysis procedure are provided in Sec. 2.4.

### 2.2 Concrete

Concrete has a low thermal conductivity and, therefore, attains temperatures at a lower rate than that of steel when exposed to a fire. Hence, concrete members are often used unprotected. Concrete is noncombustible and protects the reinforcing steel. Heat transfer calculations are carried out for concrete members to evaluate the time-dependent member temperature distribution and to obtain the unexposed surface temperatures. Such calculations are done to determine the mechanical behavior of concrete members at elevated temperatures. The following sections describe various approaches that are available to determine concrete member temperatures during a fire exposure.

For the design of concrete structures in fire, both the temperatures of concrete and the reinforcing steel need to be known. Design charts are available that provide thermal gradients in beams, columns, and slabs exposed to standard fires. Concrete temperatures can be determined by using simplified and comprehensive approaches. Simplified approaches can typically be used for 1-D heat transfer, which is usually applicable for concrete slabs or walls. For realistic fires, it is best to use comprehensive approaches for determining temperatures of 3-D structures comprising beams, columns, *etc*. The use of a particular approach may depend on the level of accuracy needed in member temperatures.

For modeling heat transfer in reinforced or prestressed concrete members, it is normally assumed that the heat transfer is mainly controlled by the thermophysical properties of concrete alone and that the temperature of the reinforcing steel is the same as that of the surrounding concrete. Although steel has a much higher thermal conductivity than that of concrete, most reinforcing steel is usually parallel to the exposure surface, and, therefore, it does not have much influence on the heat transfer that occurs perpendicular to the fire-exposed surface [17].

For concrete members, a 2-D FE program is probably the most accurate, economical, and straightforward approach to determine time-dependent temperatures of a concrete section during a fire exposure. Such an approach allows for inclusion of any combination of materials, shapes, and voids (if any).

#### 2.2.1 Simplified Approaches for Concrete

Simplified approaches for determining the heat transfer in concrete are usually applicable for the cases where the heat flow is 1-D. This is typically true for concrete slabs or walls. In some cases, simplified approaches can be extended to obtain temperatures when the heat flow is in two directions. One example of this application is shown later in this section; see Eq. (21).

Graphical representations of the temperature distribution in concrete members exposed to the standard ASTM E119 fire are available in the literature. Some of the main sources of graphical data are the Institution of Structural Engineers (ISE) and Concrete Society Design Guide (1978) [30], the International Federation for Structural Concrete (FIP/CEB) Report (1978) [31], and EN 1992-1-2 [32]. The ISE and Concrete Society Design Guide [30] provides temperature profiles for both flat soffit slabs and beams when exposed to the standard furnace fire. The document recommends that the temperatures for lightweight concrete beams may be taken as 80% of those for normal-weight concrete. The FIP/CEB Report (1978) [31] gives temperature data on different types of concrete but only for exposure to the standard fire. Annex A of EN 1992-1-2 [32] provides temperature profiles for slabs and beam–column sections of normal-weight concrete. These values are from calculations. It is not clear if these temperature profiles were calibrated against suitable test data. In addition, the thermal properties of concrete that were used in this calculation are not known.

**Fig. 9:**
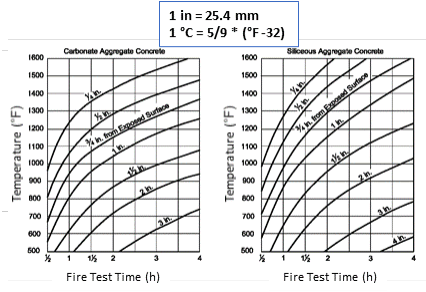
Temperatures within normal-weight concrete slabs or panels during ASTM E119 fire exposure [33] (reprinted with permission).

**Fig. 10:**
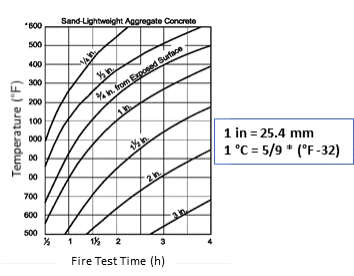
Temperatures within sand-lightweight concrete slabs or panels during ASTM E119 fire exposure [33] (reprinted with permission).

Figures 9 and 10 show plots of temperature as a function of time at different depths into the member for three different aggregate types: siliceous, carbonate, and sand-lightweight [33]. Graphs are available for determining temperatures at unexposed surfaces of concrete members for various thicknesses and aggregate types (ACI 216.1-07). These graphs were also developed using the ASTM E119 standard fire as the fire exposure curve.

**Fig. 11:**
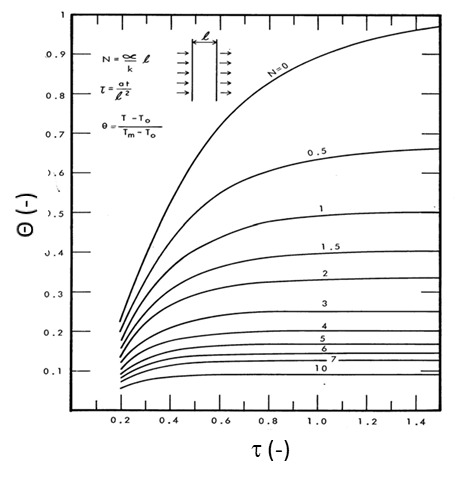
Graphical heat transfer solution for ASTM E119 fire exposure of concrete slab [20] (reprinted with permission).

Lie [20] conducted a 1-D analysis of the evolution of dimensionless temperatures in concrete slabs or walls exposed to ASTM E119 fire exposure. He developed a series of graphs based on a heat transfer analysis. He suggested using values of thermal properties evaluated at mean temperature when these properties varied with temperature. He proposed the use of principles of superposition to problems involving 2-D and 3-D geometries. Figure 11 is a graph taken from Lie’s work.

In 1980, the Concrete Reinforcing Steel Institute (CRSI) compiled all available information pertaining to the fire resistance of concrete exposed to ASTM E119 fire. It outlined empirical design procedures and described performance of a real structure in an actual fire by discussing effects of fire on the capacity of concrete elements [34].

Malhotra [12] described design methods for fire performance of reinforced concrete. His design procedure included data for strength of concrete and steel and temperature distribution charts obtained from exposure of concrete specimens to the ISO 834 fire exposure curve.

Munukutla [35] developed a 1-D numerical model for computing the transient temperature profile in a concrete wall/slab exposed to fire from one side. Temperatures through the thickness of the wall/slab were computed using an FD method. Temperature-dependent thermophysical properties were included.

Most empirical methods are either based on a solution to Fourier’s heat flow equation or obtained from the use of curve fitting of temperature data from furnace tests. There are two widely used methods: one introduced by Wickstrom [36, 37] and the other presented by Hertz [38]. Wickstrom [37] developed the following empirical method for normal-weight concrete members based on his computer-based thermal analysis procedure using TASEF-2 software. For exposure of a concrete slab to the standard fire [1, 2], the following equations were determined:

$T_{w}=\;\eta_{w}T_{f}\;\;\;\;\;\;\;\;\;\;\;\;\;\;\;\;\;\;(17)$

$T_{c}=\;\eta_{x}T_{w}\;\;\;\;\;\;\;\;\;\;\;\;\;\;\;\;\;\;(18)$

where

$\eta_{w}=\;1\;-\;0.062\cdot t_{h}^{-0.88}\;\;\;\;\;\;\;\;\;\;\;\;(19)$

$\eta_{x}=\;0.18\cdot \ln\left(\frac{t_{h}}{x^{2}}\right)-0.81\;\;\;\;\;\;\;\;\;\;\;\;(20)$

where $T_{w}$ is the fire-exposed surface temperature; $T_{c}$is the concrete temperature at a depth *x* (m) from the exposed surface; and $t_{h}$is the exposure time in hours. This method can also be applied for heat flow at corners of beams, where the heat flow is in two directions. In this case, $\eta_{y}$can be obtained using an expression similar to that shown in Eq. (20). Wickstrom proposed the following equation for concrete temperatures when the heat flow is in two directions:

$T_{c}=\;\left[\eta_{w}\left(\eta_{x}+\eta_{y}-2\eta_{x}\eta_{y}\right)+\eta_{x}\eta_{y}\right]{\;T}_{f}\;\;\;\;\;\;(21)$

Wickstrom also discussed how these equations can be modified for other types of concrete. He also demonstrated how such equations can be applied in the case of realistic fires with a decay period. One shortcoming of this approach is that it does not address different rates of temperature increases in wider or narrower beams. Note that this simple approach is valid for normal-weight concrete with a water content of 1.5% exposed to the ISO 834 fire and is not accurate in the fire decay period because the maximum concrete temperature is reached much later than when the fire reaches its peak temperature.

In Hertz’s approach [38], the time-dependent, unidirectional temperature rise in the member temperature is given by:

$∆T\left(x,t\right)=f_{1}\left(x,t\right)+\;f_{2}\left(x,t\right)+f_{3}\left(x,t\right)\;(22)$

where the functions *f_1_*, *f_2_*, and *f_3_* are obtained from solutions of heat transfer equations that are subjected to specific boundary conditions. These functions and the limitations are discussed in Ref. [38]. For 2-D heat flow, the above equation can be modified as discussed in Ref. [4]. Purkiss [4] provided a comparison of the surface temperatures predicted by Wickstrom, Hertz, and EN 1992-1-2, Fig. A2 therein [32]. This showed that all temperatures are within 40 °C of each other. Therefore, any of these approaches can be used for all practical purposes.

In structural fire engineering, concrete fire resistance is often described by the concept of the 500 °C isotherm method. This was first introduced in Ref. [24] and then introduced in Eurocode 2-1-2 (EN 1992-1-2:2004) [32] and a CEB-FIP bulletin [39]. This is based on the observation that the reduction of concrete strength is not significant below 500 °C, and the strength decreases radically beyond this temperature, eventually reaching about 30% of its ambient temperature strength at 700 °C. Following the approach of Wickstrom [36, 37], considering uniaxial heat flow, the position for a temperature rise of *ΔT_x_* at time *t* and furnace temperature rise of *ΔT_f_* is given by the following equation:

$x={\left[\frac{\frac{\alpha }{0.417.10^{-6}}}{exp\left(4.5+\frac{∆T_{x}}{0.18\eta_{w}∆T_{f}}\right)}\right]}^{0.5}\;(23)$

where *α* is the thermal diffusivity in m^2^/s, and temperatures are in °C. Other simple calculation approaches require determining the exposed surface temperature and applying the principles of transient thermal diffusion through the thickness of a member. For slabs/walls, simple 1-D heat conduction equations can be used to obtain the temperatures. Munukutla [35] proposed that the exposed surface temperatures of concrete walls can be approximately taken as 0.85 times the furnace gas temperatures. This approximation can be questioned, since it is clear that surface temperatures will vary based on fire conditions and types of concrete materials used. This empirical technique cannot be applied to a slab element because, according to BRANZ test data [40], it is apparent that a slab does not follow the same thermal relationship, and, therefore, a more exact calculation technique is needed.

Kodur *et al*. [41] demonstrated that current design graphs and simplified approaches do not produce reliable temperatures in rebar and concrete. They proposed a simplified approach for estimating cross-sectional member temperatures during a fire exposure. They conducted numerous FEA simulations by varying section geometry, concrete characteristics, and fire exposure conditions and developed a statistical regression analysis–based approach to generate equations for temperature variations in members. The following equations were derived for 1-D and 2-D heat transfer (which follow the form of Wickstrom’s equations). Note that for 2-D heat flow, the temperatures are obtained by using the heat flow from each side of the exposure:

1-D:

$T_{z}=c_{1}.\eta_{z}.\left(a\;t^{n}\right)\;(24)$

$\eta_{z}=a_{1}.\;ln\frac{t}{z^{1.5}}+a_{2}.\sqrt{z}+a_{3}\;(25)$

2-D:

$T_{z}=c_{2}.\left[{b_{1}.\eta }_{z}.\eta_{y}+\;b_{2}\left(\eta_{z}+\eta_{y}\right)+b_{3}\right].\left(a\;t^{n}\right)\;(26)$

where $\eta_{y}$ is calculated in the same way as for $\eta_{z}$. Here, $b_{1}$, $b_{2}$, $b_{3}$ are obtained from regression analysis; $c_{1}$ and $c_{2}$ account for the type of concrete; and *a* and *n* are coefficients needed to describe the standard fire exposure. For example, *a* = 935 and *n* = 0.168 for the ISO 834 fire. In the work by Kodur *et al*. [41], $c_{1}$ values were found to be 1.0, 1.01, 1.12, and 1.12, and $c_{2}$ values were 1.0, 1.06, 1.12, and 1.20 for normal strength concrete with carbonate aggregate (NSC-CA), high strength concrete with carbonate aggregate (HSC-CA), normal strength concrete with silicious aggregate (NSC-SA), and high strength concrete with silicious aggregate (HSC-SA), respectively; $a_{1}$, $a_{2}$, and $a_{3}$values were 0.155, −0.348, −0.371, respectively, and $b_{1}$, $b_{2}$, and $b_{3}$ values were −1.481, 0.985, and 0.017, respectively.

Gao *et al*. [42] used a regression analysis of FEA data to obtain simple equations for temperatures in reinforced concrete beams at any point in a beam’s cross section as a function of its coordinates, beam width, and exposure time. From their FEA simulations, they determined that the temperature distribution over the midwidth vertical line of the cross section can be determined from a 1-D heat flow equation when the beam cross-sectional area is large (greater than 600 mm × 600 mm). They proposed the following equation for the temperature rise at a point along the midwidth line:

$∆T=\theta_{d,\;120}{\;k}_{t\;}k_{b}\;(27)$

where $\theta_{d,\;120}$ represents the increase in temperature at 120 min for a certain depth, *d*, and a beam width of 600 mm. Here, ${\;k}_{t\;}$is used to include the effects of fire exposure time, and the influence of beam width is included through $k_{b}$. Based on the regression analysis, $\theta_{d,\;120}$ is expressed with the following equation:

$\theta_{d,\;120}=\;a_{0}\;.\;exp\left(a_{1}\;d\right)+a_{2}\;(28)$

In the work by Gao *et al*. [42], values for the coefficients $a_{0}$, $a_{1}$, and $a_{2}$ are 872.5, 1.771 × 10^−2^, and 4.526, respectively, for siliceous aggregate concrete and 895.7, −1.881 × 10^−2^, and 1.882, respectively, for calcareous aggregate concrete. The values of ${\;k}_{t\;}$can be obtained from a Morgan-Mercer-Flodin function (MMF) [43] as shown below:

$k_{t}=\;\frac{t_{1}t_{2}+\;t_{3}\;t^{t_{4}}}{t_{2}+\;t^{t_{4}}}\;(29)$

where $t_{i}$ (*i* = 1,2,3,4) represents a function of concrete depth. This quantity can be expressed as polynomials, as shown below:

$t_{i\;\left(i=1,2,3,4\right)}=\;m_{1}+\;m_{2}\;.\;d+\;m_{3}\;.\;d^{2}+\;m_{4}\;.\;d^{3}+\;m_{5}\;.\;d^{4}\;(30)$

where coefficients $m_{j}$ (*j* = 1,2,3,4) were determined through least square analysis of FE data. Please see Ref. [42] for these values. Using curve-fitting of their FE simulated data on beam widths ranging from 200 mm to 600 mm for depths from 5 mm to 200 mm, the following expression was obtained for $k_{b}$:

$k_{b}=exp\left[b_{0}+\;\frac{b_{1}}{\frac{b}{200}}+\;b_{2}\ln\left(\frac{b}{200}\right)\right]\;(31)$

where the coefficients are expressed through the following algebraic equations:

$b_{0}=\;-0.1307-1.45\;.\;10^{-2}d+5.809\;.\;10^{-5}d^{2}\;(32)$

$b_{1}=\;-0.1712-2.035\;.\;10^{-2}d-\;3.421\;.\;10^{-5}d^{2}\;(33)$

$b_{2}=\;7.388\;.\;10^{-2}+6.593\;.\;10^{-5}d-4.116\;.\;10^{-5}d^{2}\;(34)$

They also proposed temperatures in corner regions based on their regression analysis of FE computed thermal data. As a verification exercise, time-temperature data from the regression equations were compared against numerical values from the FE model. Good agreements were shown in the plots. They also compared these plots with test data from Wu *et al.* [44] and Kodur *et al.* [41].

**Fig. 12:**
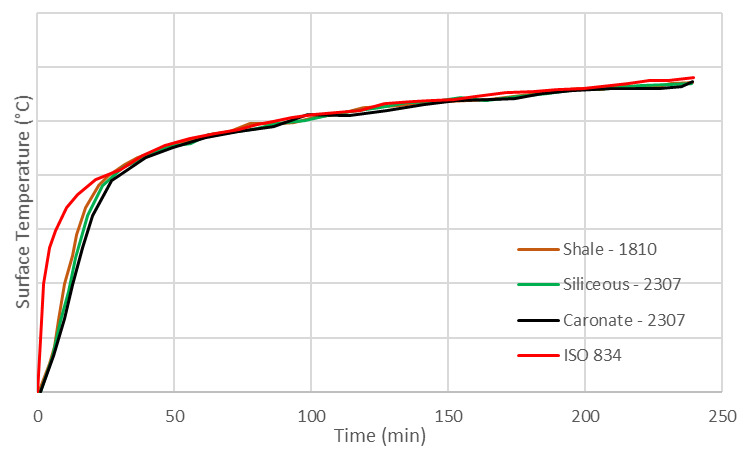
Slab exposed face surface temperature of various concrete aggregates. Adapted from Fig. 4.2 of Ref. [45].

Levesque [45] proposed the use of the “lumped heat capacity” method for obtaining the concrete temperature at the exposed surface. Although the “lumped heat capacity” is not appropriate for concrete because of its rather low thermal conductivity values, Levesque contended that this approach can still be used to obtain the exposure surface temperature. Therefore, Eq. (4) can be used to obtain the change in surface temperatures. The proper thermophysical properties of concrete and the convective and radiative heat transfer parameters are required to obtain an accurate estimate of the concrete surface temperatures. Figure 12 is a plot of surface temperatures of various concrete materials exposed to the standard fire.

The 1-D heat conduction can be used to obtain temperatures within a member when exposed to a fire temperature at one end. The following heat conduction equation needs to be solved (note that the equation is written for the case of constant thermophysical properties for a member):

$\frac{\partial T}{\partial t}\;=\;\alpha \frac{\partial^{2}T}{\partial x^{2}}\;\;\;\;\;\;\;\;\;\;\;\;\;\;\;\;(35)$

where *T* is the temperature of the member at a given position, *t* is the time, *x* is the distance along the heat flow direction, α is the thermal diffusivity of the member, and $\alpha \;=\;k/(\rho C_{p})$, where *k* is the thermal conductivity, ρ is the density, and $C_{p}$ is the heat capacity.

The above equation needs to be solved with appropriate initial and boundary conditions. The proper choice of initial and boundary conditions is necessary for determining the member temperature accurately. For a slab exposed to fire temperature (*T_f_*), a boundary condition obeying Newtonian heating/cooling can be used at the exposed surface ($h_{eff}$ is the effective heat transfer coefficient in fire):

$h_{eff}\left(T_{f}-T\right)\;=\;-k\frac{\partial T}{\partial x}\;\;\;\;\;\;\;\;\;\;(36)$

When the member is exposed to a constant fire temperature, Heisler charts [18] provide graphical solutions to the temperature fields. These charts plot dimensionless temperatures (*θ*) as functions of the dimensionless Fourier number (*Fo*) and the Biot number (*Bi*) as defined below:

$\theta \;=\;\frac{T-T_{f}}{T_{i}-T_{f}}\;\;\;\;\;\;\;\;\;\;\;\;\;\;\;\;(37)$

**Fig. 13:**
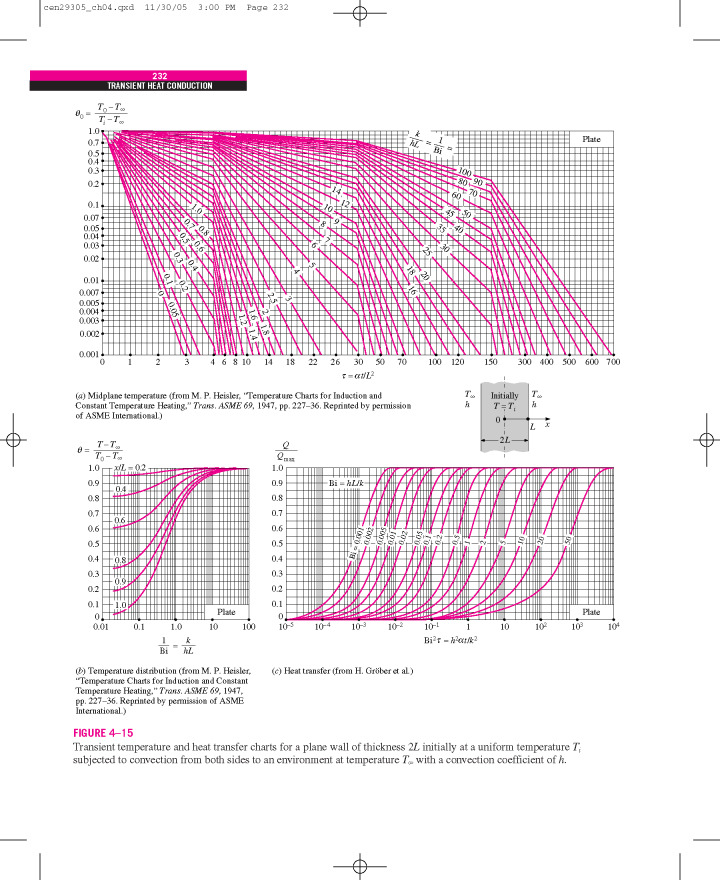
Transient temperature at the center plane of a slab subjected to convective heat exchange at both boundary surfaces [18] (reprinted with permission).

**Fig. 14:**
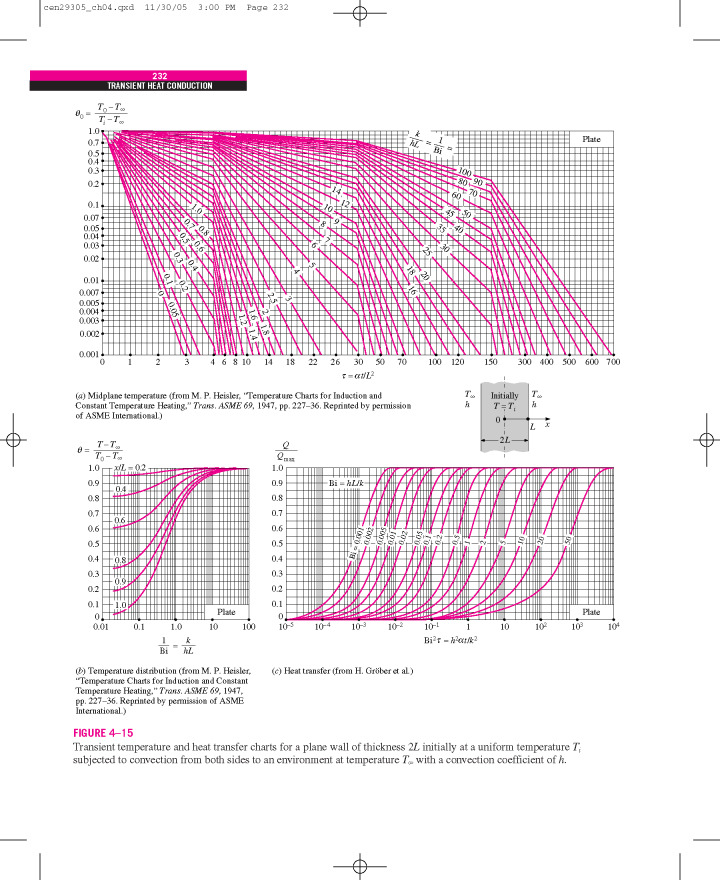
Position correction chart for use with Fig. 13 [18] (reprinted with permission).

$Fo\;=\;\frac{\alpha t}{L^{2}};\;\;Bi=\frac{h_{eff}L}{k}\;\;\;\;\;\;\;\;\;\;\;(38)$

where *T_i_* is the initial member temperature, *L* is the member length, and *k* is the thermal conductivity. Solutions to Eq. (35) subject to the boundary condition in Eq. (36) have been solved for a wide range of values for the Biot and Fourier numbers and are compiled in Heisler charts (see Figs. 13 and 14).

One major problem with the above approach is that the fire temperature, *T_f_*, is assumed to be constant for the duration of the fire. This is not the case in reality. Fire temperature varies throughout the duration of the fire in a real fire event. Lie [20] circumvented this problem by considering the average value of an ISO fire curve as the constant fire temperature during the entire duration of fire. Lie developed Fig. 11 using variations of the methodology discussed in developing the Heisler charts (Fig. 13 and 14).

##### 2.2.1.1 Simplified Analytical Model for Computing Concrete Temperatures in 1-D Heat Transfer

The following approach is valid for 1-D heat flow in a concrete slab under certain conditions [46]. The analytical equation is rather simple for 1-D heat transfer in a semi-infinite solid with Dirichlet type of boundary conditions. (Note that the Dirichlet or the first-type boundary condition specifies the values a solution needs to take on the boundary of the domain, *e.g.*, temperature in this case.) If the concrete slab is assumed to be a semi-infinite body in the direction of its depth, then a simple expression can be used to describe the thermal field as shown below. A thick body can be modeled as a semi-infinite solid if we are interested in the variation of the thermal field near one surface, and the temperature at the far end does not change over time from the initial values. Note that similar analytical equations can be developed for other boundary conditions [47].

**Fig. 15:**
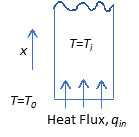
Schematic representation of 1-D heat flow in a concrete slab (semi-infinite solid) along its depth.

Figure 15 shows a schematic representation of 1-D heat flow in a semi-infinite solid (concrete slab). For the concrete slab to be semi-infinite, the unexposed surface temperature should not change over time during the fire exposure. This is a reasonable assumption for the case of a composite floor system exposed to a fire underneath the slab. In other words, the concrete slab is assumed to be thermally thick. The following inequality must hold for the semi-infinite solution to apply [47]:

$\frac{x}{\sqrt{4\alpha t}}\;\geq \;0.5\;(39)$

where *x* is the thickness of the slab, *t* is the time, and *α* is the thermal diffusivity. Equation (35) is valid for 1-D transient heat flow assuming that the thermophysical properties are independent of temperature.

The boundary and initial conditions are:

At time *t* = 0, *T*(*x*,0) = *T_i_*

At time *t* > 0, *T*(0,t) = *T_0_*

where *T_i_* is the initial temperature, and *T_0_* is the temperature at the exposed surface (*e.g.*, fire temperature). The solution of Eq. (35) subject to the above initial and boundary conditions is given by the following equation, obtained using a Laplace-transform technique:

$\frac{T(x,t)-T_{0}}{T_{i}-T_{0}}\;=\;erf\left(\frac{x}{2\sqrt{\alpha t}}\right)\;(40)$

where the error function (*erf*) is defined as:

$erf\left(\frac{x}{2\sqrt{\alpha t}}\right)\;=\;\frac{2}{\sqrt{\pi }}\int_{0}^{x/2\sqrt{\alpha t}}e^{-\eta^{2}}d\eta \;(41)$

where *η* is a dummy variable.

Clearly, the assumption of a constant fire exposure temperature is a major impediment toward using such simple methods for determining transient member temperatures as a function of location. For accurate determination of temperatures, the solution to Eq. (35) needs to be obtained considering proper initial and boundary conditions such as the time-dependent fire temperatures at the exposed surface of the member. There are several analytical approaches available for obtaining expressions for the case of time-dependent fire temperatures. Some of them are the Laplace transform, integral method, Duhamel’s theorem, *etc*.

Laplace transform techniques are described in any advanced textbook on heat transfer. They are not described here. The integral method, Duhamel’s theorem, and energy-based approach are described briefly below.

##### 2.2.1.2 Integral Method

The integral method is an approximate method for obtaining the solution of the transient, nonlinear heat conduction equations. The use of this method was first introduced by Goodman [48]. This method is based on the concept that the solution will satisfy the problem on the *average* over the region considered. The method comprises the following steps [48]:

1. The differential equation of heat conduction is integrated over a thermal layer, *δ(t)*. This layer is much like the boundary layer concept used in fluid mechanics. This layer is defined as the distance beyond which the region is unaffected by the applied boundary condition, and, hence, there is no heat flow in the region beyond *δ(t)*. The thermal layer thickness changes with time in transient heat conduction problems.
2. A suitable profile is assumed for the temperature field in the thermal layer. A fourth-order polynomial approximation is often used, for which coefficients are determined from the application of the initial and boundary conditions and from the definition of the thermal layer as stated above.
3. The temperature profile is then substituted into the heat balance equations. When integrations are performed with respect to the space variable, an ordinary differential equation for the thermal layer thickness, *δ(t)*, is obtained. Solution to this equation, subject to the initial condition, provides an equation for the evolution of the thermal layer as a function of time.
4. Substitution of the expression of the thermal layer thickness into the equation for the temperature (obtained in step 2) results in an expression for the temperature field as a function of the space variable and time.

This method allows for computation of the temperature profile within the thermal layer. Temperatures for the region beyond the thermal layer remain at initial temperature. When the thermal layer reaches the thickness of the specimen, the thermal layer does not have a physical significance anymore. Then, the integral method equations are derived with the finite thickness of the specimen using procedures explained previously for the thermal layer case. Reference [48] provides a few simple examples for applying this approach for solving 1-D heat flow equations subject to several boundary conditions.

##### 2.2.1.3 Duhamel’s Theorem

Duhamel’s theorem (also known as the Duhamel superposition integral) showed that the solution of the heat conduction problem with time-dependent boundary conditions can be related to the solution of the heat conduction problem with time-independent boundary conditions. This integral shows that the problem of a time-dependent boundary condition could be reduced to that of a stepwise boundary condition. Duhamel’s theorem can be described by using a simple example as follows.

A body is assumed to be at an initial temperature of, say, zero degrees Celsius for a period *t* = *s*, when it is suddenly exposed to a unit change in a boundary condition (*e.g.*, disturbance, *D*), following which its temperature changes as shown below:

φx,t =0,       t<sψ(x,t-s),​​​​ t>s           (42)

where ψ(x,t) is the temperature resulting from a unit change in the disturbance, and $\varphi \left(x,t\right)$ is the resultant temperature. If the boundary condition changes as a continuous function of time (instead of being a stepwise change), then the following procedure can be adopted. First, the disturbance is approximated by assuming that it is suddenly changed to *D*(*0*) when *t* = *0* and is kept at this value until *t* = *s_1_*, when it is suddenly changed by an amount of *D*(*s_1_*) – D(*0*) and held at this value until *t* = *s_2_*, and so on and so forth. Then, the temperature after time *t* = *s_n_* can be written as:

$\varphi \left(x,t\right)\;=\;D(0)\psi (r,t)+[D(s_{1}-D(0)]\psi (r,t-s_{1})$

$\;\;\;+[D(s_{2}-D(s_{1})]\psi (r,t-s_{2})$

$\;\;\;\;\;\;+....+[D(s_{n}-D(s_{n-1})]\psi (r,t-s_{n})\;(43)$

Define:

$\;\;\;D\left(s_{m}\right)-D\left(s_{m-1}\right)=\Delta D_{m}\;,\;s_{m}-s_{m-1}=\Delta s_{m}\;\;\;\;\;\;\;\;\;\;(44)$

Then, Eq. (43) can be written as:

$\varphi \left(x,t\right)=D\left(0\right)\;\psi \left(x,t\right)+\sum_{m=1}^{n}\psi \left(x,t-s_{m}\right){\left(\frac{\Delta D}{\Delta s}\right)}_{m}\Delta s_{m}\;\;\;\;\;\;(45)$

In the integral form, Eq. (45) can be written as follows (when n→∞):

$\varphi \left(x,t\right)=D\left(0\right)\;\psi \left(x,t\right)+\int_{0}^{t}\psi \left(x,t-s_{m}\right)\frac{dD\left(s\right)}{ds}ds\;\;\;\;\;\;\;\;(46)$

See Ozisik [47] for further details on this technique. This theorem is applicable to: (1) linear equations; (2) the case when the initial temperature is zero. If it is not, then a new variable needs to be defined, (*T − T_i_*), where *T_i_* is the initial temperature. See Ozisik [47] for solutions of a few example problems employing this approach. Appendix A shows two examples using this approach.

##### 2.2.1.4 Energy-Based Approach

Panedpojaman [49] proposed an energy-based approach for determining concrete section temperatures when they are exposed to fire on one side. This approach simplifies the FD approach for heat transfer and is based on an energy conservation principle and a predetermined function for the shape of the temperature profile through a concrete section. The energy input due to fire exposure is equal to the sum of the energy used to raise the concrete temperatures and the energy lost at the unexposed surface. The author proposed a power-law function for expressing the temperature as a function of the distance from the exposed surface. The author varied the exponent of the power-law function in order to match the experimental results.

Although this approach is simple and can be used in a spreadsheet, its application is limited. This is because the power-law exponent is an unknown quantity, and it needs to be determined on a case-by-case basis. It may be mentioned here that there are no simplified approaches that deal with concrete spalling during fire. However, there are comprehensive models that deal with concrete spalling (see Sec. 2.2.2).

#### 2.2.2 Comprehensive Approaches for Concrete

The advanced calculation approaches provide a more realistic analysis of real concrete structures exposed to a fire. This is the only option available for studying the effect of fire in a real structure comprising members with complex geometries, multiple materials, connections, *etc*. Also, comprehensive approaches need to be used for describing heat transfer in 2-D or 3-D. This is true for heat transfer in beams, columns, *etc*.

Computer-based analysis methods have been frequently used to predict concrete temperatures in fire. These models range from simple 1-D models to advanced 3-D FE and FD models. Lie and Allen [50] developed an FD model to study the heating behavior of circular reinforced concrete columns exposed to ASTM E119 fire. Similar models have been developed for floor slabs [51] and reinforced concrete columns [52].

Ahmad and Hurst [53, 54] proposed a 1-D FD analysis of carbonate and siliceous concrete slabs. They considered coupled heat and mass transfer. Both dehydration/evaporation and changes in porosity were considered.

Some of the heat transfer software/algorithms that have been used to study the evolution of concrete temperatures include:

(1) HEATING7 [55],
(2) FIRES-T3 [25],
(3) TASEF-2 [23, 56],
(4) HEAT [35],
(5) SAFIR [46], and
(6) general purpose FE software, *e.g.*, ABAQUS, ANSYS, MSC-NASTRAN, and COMSOL [26–29].

The scientific capabilities of these commercial software products vary, and some of them may not include complex phenomena that occur at elevated temperatures (*e.g.*, spalling). Most commercial software should be able to handle time-dependent boundary conditions and material properties. More advanced models include moisture transport and pore pressure analysis. These advanced models should include material models for nonhomogeneous bodies and be able to predict spalling during fire.

Heat transfer analysis of composite floor systems in fire has been conducted by researchers in recent years [57, 58]. Heat flow in both steel beams and concrete slabs must be modeled to accurately determine the thermal behavior of composite floor systems. Alfawakhiri *et al*. [57] performed a heat transfer analysis of the composite floor exposed to fire in the first Cardington test [59] using the SAFIR software [46]. The SAFIR program allows for including concrete thermal properties as a function of the moisture level. They neglected the profiled metal deck and used an average slab thickness in their model. The simulated time-temperature data matched measurement data both at the steel beams and at most locations in the concrete slab. However, in the concrete slab, the predicted temperatures were higher than those obtained in experiments, especially at locations close to the exposed surface. This discrepancy was attributed to the following phenomena that were not included in the model: (1) the separation of the profiled steel deck from the concrete slab, thereby leading to the slowing of heat transfer across the exposed surface of concrete slab, and (2) variation of the moisture level as a function of location and local temperature in the slab.

Lamont *et al.* [58] modeled the heat flow in the composite steel and concrete slab used in the Cardington tests [59]. They used an adaptive 2-D FE program called HADAPT to conduct nonlinear, transient heat flow analysis. This program models evaporation of moisture from pores in a concrete slab by assuming that a phase change occurs at 100 °C. The phase change was modeled using an enthalpy method. However, their model did not include moisture migration in concrete. The separation of the profiled metal deck from the concrete slab was included by using “interface elements.” The heat transfer across the steel metal deck/concrete slab interface was modeled using a Newtonian heating/cooling approach, which was implemented as a constraint in the discretized heat transfer equations. The interface elements were generated using coincident nodes across the faces of steel and concrete regions. They modeled the heat transfer in the first three Cardington tests by (1) including the profiled metal deck and (2) excluding the profiled metal deck. Their computed results showed that the model including the profiled metal deck overpredicted the steel temperatures but accurately predicted the concrete slab temperatures. Overprediction of steel temperatures was attributed to the inadequacies in modeling moisture migration and evaporation in concrete. Concrete slab temperatures in the three Cardington tests were modeled satisfactorily. Reference [60] describes the use of a comprehensive FE approach in modeling the effects of fire (standard fire) on a composite floor system.

### 2.3 Calculation of Temperatures in Concrete-Filled Steel Tubes (CFSTs)

The composite structure formed by using a steel profile with concrete has many advantages such as better load capacity, stiffness, and durability under fire exposure. Eurocode 4 (EN1994-1-2 [14]) provides guidelines for the determination of the temperature in the cross section of composite columns under fire exposure, but it does not address CFSTs. A detailed FE analysis with appropriate thermal boundary conditions and temperature-dependent thermal properties may be necessary to get an accurate description of the temperature field. Proper values of thermal interface conductance at the steel/concrete interface are needed. An air gap forms at the steel/concrete interface during the fire-induced heating process that provides resistance to heat flow at the interface. Hu *et al.* [61] suggested that the steel emissivity should be a temperature-dependent property and that a value of 0.38 for furnace or fire emissivity could be used. Ghojel (2004) [62] proposed the following equation for the thermal interface conductance, $h_{j}$:

$h_{j}=336.9-268.1\;exp\left(-18.2\;T_{s}^{-0.845}\right)\;(47)$

where *T_s_* is the steel temperature. In his tests, he observed that moisture migrated toward the interface as specimen temperature increased. Hu *et al*. [61] observed that Ghojel’s equation was based on a limited number of tests and did not include the influence of gas pressure at the steel/concrete interface. They subsequently modified Ghojel’s equation to address these two shortcomings as shown below for circular columns:

$h_{j}=\mu \left[160.5-63.8\;exp\left(-339.9\;T_{s}^{-1.4}\right)\right]\;(48)$

Based on numerical fitting, a value of 0.8 was proposed for *μ* for high-strength CFST columns. Eurocode EN 1994-1-2 [14] described methods for calculating the fire resistance of CFST columns. Espinos *et al*. [63] demonstrated a simple calculation approach for evaluating the fire resistance of unreinforced CFSTs. It is desirable to have a uniform equivalent temperature for the whole concrete core and another one for the steel tube, which follows recommendations in EN 1994-1-2. Espinos *et al*. [63] demonstrated a plastic resistance approach and a flexural stiffness approach to obtain the equivalent temperature of the concrete core, which is the maximum temperature from these two approaches based on a regression analysis:

$T_{c,\;eq}=\;-186.4+5.76R-0.03R^{2}+22.6\frac{F}{V}-0.32{\left(\frac{F}{V}\right)}^{2}+0.14R.\frac{F}{V}\;(49)$

where *R* is the fire resistance (in minutes). The following expression was obtained for the equivalent temperature of steel:

$T_{s,\;eq}=\;342.1+10.77R-0.044R^{2}+3.922\frac{F}{V}-0.025\;R.\frac{F}{V}\;(50)$

Rodrigues and Moreno [64] obtained similar expressions for the equivalent temperatures based on their regression analysis of numerical parametric simulation. However, they did not include thermal resistance at the steel/concrete interface. Such methods of obtaining equivalent temperatures are attractive to designers because they do not need to make advanced numerical heat transfer analysis to obtain the temperatures.

### 2.4 General Procedures for Numerical Modeling of Heat Flow

#### 2.4.1 Geometry Building and Meshing

The geometry includes structural members such as steel, concrete, *etc*. In most software, an electronic data interface is provided to retrieve the geometry. Typically, the solid geometry is built using computer-aided design (CAD) software. Meshing is important because the accuracy of calculated results depends on the quality of the mesh. In general, higher mesh density results in higher computational accuracy and requires more central processing unit time (as storage and memory requirements increase).

#### 2.4.2 Description of Material Properties

The material properties are assigned to a region comprising cells or elements based on the material type associated with that region. Some of the typical material properties include thermal conductivity, enthalpy, emissivity, density, latent heat (if applicable), heat capacity, *etc*. Some of these properties could be functions of temperature. Some commercial software have a built-in materials database consisting of properties of common materials.

#### 2.4.3 Assigning Initial and Boundary Conditions

For a typical heat transfer analysis, initial temperatures in the members need to be provided. These can be provided in terms of constant values or a distribution of values for a material domain. Different boundary conditions are applied at different locations. For example, at the exposed surface, a heat flux, or a convective heat flow condition or a radiation condition, can be prescribed. Typically, the Dirichlet condition or convective heat flow condition is applied to the unexposed surface.

#### 2.4.4 Selecting Control Parameters

Some of the control parameters include simulation time, time step values, convergence criteria, relaxation factors, output result frequency, *etc*. Appropriate choices of these parameters are needed to obtain accurate results in a reasonable computational time. In most software, minimum and maximum time step values are provided. For the explicit time integration approach, the maximum time step value needs to satisfy the value from the stability criterion. Since an implicit formulation is unconditionally stable, a large value of the maximum time step can be selected. Convergence criteria are needed for an iteration sequence associated with the solution of the nonlinear system of equations encountered in an implicit time integration scheme. An optimum value of the convergence criteria is needed to obtain an accurate solution in a reasonable time. The relaxation factors relate to the underrelaxation factors used in nonlinear iterations.

#### 2.4.5 Solution

In this step, the partial differential equations of heat transfer [*e.g.*, equations for the conservation of energy, *i.e.*, Eq. (16)] are solved subject to the prescribed initial and boundary conditions. Results are stored at a time frequency or steps specified by the user. Typical output quantities include the temperature field, heat flux, and temperature gradient.

#### 2.4.6 Postprocessing

Most software products have postprocessing capability that allows direct visualization of computational results. These may include contour color plots of time-dependent temperature distribution, temperature gradient, heat flux, *etc*. Nodal quantities such as time-temperature data at specific locations in the computational domain can be easily output by the software.

### 2.5 Uncertainties in Computed Temperatures

For modeling structural behavior during fire, three separate analyses are typically conducted: (1) fire propagation and growth (fire modeling), (2) transient heat transfer in structural members due to fire, and (3) structural analysis accounting for both the thermal and mechanical loads. This document deals with heat transfer analysis, or step (2). However, it is important to properly transfer thermal data from the fire model through the heat transfer model to the structural analysis models. Any inaccuracies in this transfer process can lead to inaccurate estimates of member temperatures. References [65, 66] describe commonly used approaches for transfer of thermal data among different models.

In the performance-based approach for structural design for fire, both spatial and temporal variations of temperatures need to be accurately determined. The ability to predict with high confidence the time-varying temperature profiles in structural members is extremely important. Therefore, uncertainties in member temperatures must be accurately estimated during a fire event. Uncertainties in estimates of thermophysical properties of structural members and boundary conditions can lead to considerable uncertainties in member temperatures. Also, uncertainties in the appropriate thickness of steel fireproofing and its thermophysical properties can contribute to the overall uncertainties in structural members. Degradation of fireproofing during a fire event can also be a contributing source of uncertainty. Spalling of concrete and buildup of fireproofing at member intersections during a fire event can further contribute to uncertainties in temperature. See Refs. [67, 68] for further information on estimating uncertainties in temperatures of concrete and steel members during fire. Uncertainties in measured temperatures at different locations of the members during the progression of fire can be significant, as evident in plots in Ref. [69]. Sensitivity studies (e.g., using an orthogonal full-factorial design approach [70] or an optimization software in conjunction with a finite element analysis method) can be conducted to determine which parameters (such as thermophysical properties, initial conditions, and boundary conditions) most significantly influence the thermal response of a structural member.

### 2.6 Visualization of Structural Behavior During Fire

The heat transfer analysis techniques discussed in this tutorial allow us to compute member temperatures when they are exposed to a real fire. These temperatures are then used in a separate structural analysis model to determine the nonlinear structural behavior under both thermal and mechanical loads. The fire propagation and growth model analyzes fire development, propagation, and growth. This is typically accomplished with a computational fluid dynamics (CFD) software. This is the first step in a typical structural fire analysis. The second step is the heat transfer due to the exposure to the fire. This step has been the focus of this tutorial. The third step is the structural analysis step, which determines the structural behavior due to fire exposure. Therefore, accurate modeling of structural behavior during fire would require a coupled fire modeling (with a CFD code), heat transfer, and structural analysis. However, this is challenging because computational length scales and typical elements used in each analysis are different. Therefore, a sequential analysis (*i.e.*, CFD for fire modeling followed by a heat transfer analysis and then a structural analysis) is often implemented, wherein fire effects are used in a heat transfer model as proper boundary conditions, and the temperature profile computed in the heat transfer model is included in the structural analysis along with mechanical loads. This approach inherently assumes that the fire modeling results affect the heat transfer calculation, while the reverse is not true. Similarly, it assumes that heat transfer analysis affects the structural calculation, while the structural calculation does not affect the heat transfer analysis. This can be construed as a weak coupling approach. However, it is a very practical and reasonable approach for large problems. This may not be applicable in prolonged and intense fires, where large structural deformation could cause damage to insulating materials (typically applied on steel members) and possibly impact the thermal profile in a significant manner.

However, one of the challenges faced by an engineer is how to visualize the structural behavior during fire. This is because of the three different computational time and length scales used for these three separate analysis techniques, *i.e.*, fire modeling, heat transfer analysis, and structural analysis. In order to address this challenge, an integrated visualization environment was recently created to study the interaction among fire, heat transfer, and structural deformation from a typical room fire [71, 72]. The fire, thermal, and structural data were linked with a separate 3-D visualization capability, to provide the ability to visualize in real time the thermal and structural behavior of a structural component in a room subjected to a typical fire in an immersive visualization environment (IVE). As a first example, a single beam in a room was used for the study. A sequential process was followed in which first the Fire Dynamics Simulator (FDS) program [73] was used to simulate the start and development of fire in the room. Then, a second computer program [27] was used to calculate how the gas temperature computed by the FDS program propagated into the beam. Finally, a third computer program [27] was used to compute how the beam deformed over time due to combined effects of thermal and mechanical loads. The three outputs from these computer programs were used in two separate visualization methods that were developed to display the computed results in an IVE. One visualization method was based on polygons, and the other was a graphics processing unit–based ray-traced volumetric rendering. Such an approach can help engineers to identify locations where structural failure might occur as a result of fire exposure.

## 3 Summary and Concluding Remarks

In this document, a brief review of various approaches used for determining the fire-induced temperatures of steel, concrete, and concrete-filled steel tubes is provided. It is obvious that a comprehensive 3-D FE analysis of heat transfer is most appropriate for obtaining the member temperatures when the geometry is complex and when multiple materials are considered. However, simplified approaches are attractive and find wide use in design offices and may be justified when employing a sufficient factor of safety in design.

Simplified approaches such as the “lumped heat capacity” approach can be used with good accuracy to obtain steel member temperatures in most cases. For concrete slabs or walls, the 1-D approaches reviewed in this document can be used with reasonable accuracy as long as the heat flow is truly in one direction. For concrete beams and columns exposed to heat along different directions, there are no simplified approaches that are available for determining member temperatures with good accuracy when the geometry is complex.

For simple geometries, such as plates, cylinders, *etc*., analytical solutions of heat flow in 2-D or 3-D directions can be obtained by applying heat flow solutions in each direction (obtained by any of the methods described in this document) and then combining the results using superposition principles described in any textbook on heat transfer. In this case, the final solution of temperature can be obtained as a product of the solutions obtained for each direction and function of time.

For 1-D heat flow in isolated steel and concrete members as a result of fire exposure, the simplified approaches discussed in this paper can produce a reasonable prediction of transient temperatures. However, in a building consisting of many members, including connections, advanced 3-D FEA models are probably the only reasonable approach for predicting temporal and spatial distributions of temperatures.

Further research efforts are needed to obtain validated simplified equations for describing heat flow in concrete members (*e.g.*, beams, columns, *etc*.) when the geometry is complex and when heat flow induced by fire exposure can occur in any direction.

One major challenge in accurate heat transfer analysis is how to determine the time-dependent temperatures at the exposed surface precisely when there is a real fire with wide variation in gas temperatures and smoke levels. This becomes more complicated when the building design has an open floor plan with a wide variation in layout, ventilation, and distribution of combustible materials such as furniture and other furnishings. This document also provides a brief introduction into the determination of uncertainties in computed temperatures and the visualization of structural behavior during fire.

## 4 Appendix A (Example of the Use of Duhamel’s Theorem)

### 4.1 Example 1

In this example, a semi-infinite solid is initially at temperature *T_i_*. At time *t* = 0, the surface at *x* = 0 is subjected to a time-dependent temperature, *e.g.*, *T(0, t)* = *f(t)*. In order to use Duhamel’s theorem, the reduced problem is first solved where the surface is exposed to a fixed temperature of *T_0_*. The solution to this problem is obtained from a standard textbook on heat conduction as:

$\frac{T(x,t)-T_{i}}{T_{0}-T_{i}}\;=\;1-erf\left(\frac{x}{2\sqrt{\alpha t}}\right)\;\;\;\;\;\;\;(A.1)$

The response to a unit step applied at time *t* = 0 is:

$\varphi \left(x,t\right)\;=\;1-erf\left(\frac{x}{2\sqrt{\alpha t}}\right)\;\;\;\;\;\;\;(A.2)$

Duhamel’s theorem applies to the general problem where (*T_0_ − T_i_*) is applied at time *t* = 0, but it is allowed to vary in time. Following Eq. (28), the solution is given by:

$T\left(x,t\right)-T_{i}\;=\;\int_{0}^{t}f\left(\tau \right)\;\frac{\partial }{\partial t}\left[1-erf\left(\frac{x}{2\sqrt{\alpha \left(t-\tau \right)}}\right)\right]d\tau \;\;\;\;\;(A.3)$

From the definition of the error function, the following expressions can be written:

$erf(\eta )\;=\;\frac{2}{\sqrt{\pi }}\int_{0}^{\eta }\exp(-\eta^{2})d\eta \;;\;\frac{d}{d\eta }(erf(\eta ))=\frac{2}{\sqrt{\pi }}\exp(-\eta^{2})\;\;\;\;\;\;\;(A.4)$

Define:

$\eta \;=\;\frac{x}{2\sqrt{\alpha (t-\tau )}}\;;\;d\eta \;=\;\frac{x}{4\sqrt{\alpha }\sqrt{(t-\tau {)}^{3}}}d\tau \;\;\;\;\;\;\;\;\;(A.5)$

Then, the solution in Eq. (A.3) can be written as:

$T\left(x,t\right)-T_{i}\;=\;\int_{0}^{t}f\left(\tau \right)\;\frac{2}{\sqrt{\pi }}\exp(-\eta^{2})\frac{\partial \eta }{\partial t}d\tau \;\;\;\;\;\;\;\;\;(A.6)$

The above equation can be rearranged as:

$T\left(x,t\right)-T_{i}\;=\;\frac{x}{\sqrt{4\alpha \pi }}\int_{0}^{t}f\left(\tau \right)\;\frac{1}{\sqrt{(t-\tau {)}^{3}}}\exp\left(-\frac{x^{2}}{4\alpha (t-\tau )}\right)d\tau \;\;\;\;(A.7)$

By rearranging the above equation, the following equation can be written for the solution:

$T\left(x,t\right)=T_{i}\;+\;\frac{2}{\sqrt{\pi }}\int_{\frac{x}{2\sqrt{\alpha t}}}^{\infty }f\left(t-\frac{x^{2}}{4\alpha \eta^{2}}\right)\;\exp\left(-\eta^{2}\right)d\eta \;\;\;\;\;\;\;\;(A.8)$

### 4.2 Example 2

**Fig. A.1:**
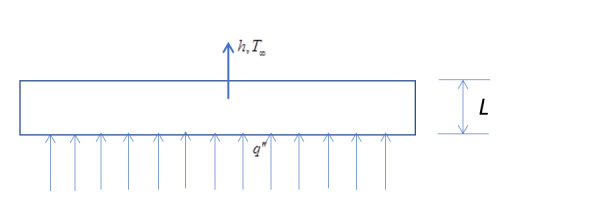
1-D heat flow in a plate.

In this example, a plate of thickness *L* (initially at ambient temperature) is subjected to a uniform heat flux at its bottom surface, and heat is lost from the top surface due to convection. The plate thickness is much smaller compared with its other dimensions. Therefore, heat lost from the sides is neglected. Assuming 1-D heat flow along the plate depth, the following heat balance equation can be written (see Fig. A.1):

$\rho_{s}c_{s}L\frac{dT}{dt}=\;q^{\prime \prime }-h\left(T-T_{\infty }\right)\;\;\;\;\;\;\;\;\;\;\;\;\;\;\;\;\;(A.9)$

with the initial condition $\;T\left(0\right)=T_{\infty }\;\;\;\;\;\;\;\;\;\;\;\;\;\;\;\;\;\;\;\;(A.10)$

The solution is obtained by integration as follows (*m* = *h/ρ_s_c_s_L*):

$T\left(t\right)-T_{\infty }=\frac{q^{\prime \prime }}{h}.\left(1-e^{-mt}\right)\;\;\;\;\;\;\;\;\;\;\;\;\;\;\;\;\;\;\;\;\;(A.11)$

In the above derivation, the heat flux, $q^{\prime \prime }$, is assumed to be constant. If the heat flux varies with time, then the solution shown in Eq. (A.11) is not straightforward. In that case, Duhamel’s theorem can be used to obtain the solution as shown below. It is assumed that the heat flux oscillates as $q^{\prime \prime }$*cos(*ω*t)*, where ω is the angular frequency. Therefore, the time-dependent disturbance is given as: D(t)=cos⁡(ωt). Using Duhamel’s theorem, Eq. (46), the solution is derived as follows:

$\frac{\varphi (t)}{q^{\prime \prime }/h}=1-e^{-mt}-\omega \int_{0}^{t}[1-e^{-m(t-s)}]\sin(\omega s)ds\;\;\;\;\;\;\;\;\;\;(A.12)$

The following solution is obtained after integration and rearrangement ($\alpha =\tan^{-1}(\omega /m)$):

$\frac{\varphi (t)}{q^{\prime \prime }/h}=\frac{m}{(m^{2}+\omega^{2}{)}^{1/2}}\cos(\omega t-\alpha )-\frac{m^{2}e^{-mt}}{\left(m^{2}+\omega^{2}\right)}\;\;\;\;\;\;\;\;\;\;(A.13)$
